# Identification of HIVEP2 as a dopaminergic transcription factor related to substance use disorders in rats and humans

**DOI:** 10.1038/s41398-019-0573-8

**Published:** 2019-10-04

**Authors:** Juan Zhao, Chunnuan Chen, Richard L. Bell, Hong Qing, Zhicheng Lin

**Affiliations:** 1 School of Life Science, Beijing Institute of Technology, 100081 Beijing, China; 2 Laboratory of Psychiatric Genomics, McLean Hospital, Belmont, MA 02478 USA; 3 Department of Neurology, the Second Affiliated Hospital of Fujian Medical University, Quanzhou, Fujian Province, P. R. China; 4 Department of Psychiatry, Institute of Psychiatric Research, Indiana University School of Medicine, Indianapolis, Indiana 46202 USA

**Keywords:** Molecular neuroscience, Addiction

## Abstract

Playing an important role in the etiology of substance use disorder (SUD), dopamine (DA) neurons are subject to various regulations but transcriptional regulations are largely understudied. For the first time, we report here that the Human Immunodeficiency Virus Type I Enhancer Binding Protein 2 (HIVEP2) is a dopaminergic transcriptional regulator. HIVEP2 is expressed in both the cytoplasm and nuclei of DA neurons. Therein, HIVEP2 can target the intronic sequence GTGGCTTTCT of *SLC6A3* and thereby activate the gene. In naive rats from the bi-directional selectively bred substance-preferring P vs -nonpreferring NP rat model of substance abuse vulnerability, increased gene activity in males was associated with the vulnerability, whereas decreased gene activity in the females was associated with the same vulnerability. In clinical subjects, extensive and significant *HIVEP2-SLC6A3* interactions were observed for SUD. Collectively, HIVEP2-mediated transcriptional mechanisms are implicated in dopaminergic pathophysiology of SUD.

## Introduction

Many environmental factors can regulate dopaminergic (DAergic) activity including *SLC6A3*, the gene encoding the dopamine transporter (DAT)^1–7^. These regulations remain mechanistically poorly understood. A critical step in dissecting these mechanisms is to identify what transcription factors (TFs) target the DAergic genome. Recently, several TFs for *SLC6A3* have been cloned, including Nurr1 (NR4A2), paired-like homeobox 3 protein (PITX3), HEY1, SP1, SP3, ^AZI2^3′UTR, SRP54, and Nfe2l1^8–13^. It is postulated that HEY1 is a TF targeting the 3′UTR^14^, whereas the others appear to target 5′ promoter regions except ^AZI2^3’UTR, a long noncoding RNA (lncRNA), that regulates Intron 1 of *SLC6A3*. Among these TFs, Nurr1, but not PITX3, is implicated in Parkinson’s disease^15,16^ and ^AZI2^3′UTR is implicated in substance use disorder (SUD)^13^. Importantly, both diseases are closely associated with altered DAergic activity. These findings indicate that identification of DAergic TFs may provide opportunities for dissecting signaling pathways that target these TFs for genomic action. This would help clarify molecular cascades underlying neuronal development, plasticity and DAergic transcription-related disorders such as SUD^17–20^.

The present study focused on delineation of new transcription mechanisms in SUD, using *SLC6A3* as a prototype target to clone DAergic TFs. Previous studies, including ours, have shown that a 121-bp fragment in Intron 1 of *SLC6A3* may display *cis*-acting activity in vitro. This 121-bp fragment served as the bait/target in our search for new DAergic TFs related to SUD.

## Materials and methods

All DNA or RNA oligonucleotides used in this study are shown in Table 1.

**Table 1:** Oligonucleotide List (DNA and RNA)

Name	Sequence	AC
a. cDNA cloning (Figs. 3, 4b)		
HIVEP2 forward	5′-aaaaagctagcaacaaaatggacactggggacaca-3′	
HIVEP2 reverse	5′-aaaaagcggccgcatcaatgtagctgactcttttctgat-3′	
b. Plasmid construction (Fig. 4b,d)		
1-forward	5′-CTA GAG AAA GCC ACG-3′	
1-reverse	5′-TCG ACG TGG CTT TCT-3′	
2A-forward	5′-CTA GGG AGA GGC AAA-3′	
2A-reverse	5′-TCG ATT TGC CTC TCC-3′	
2B-forward	5′-CTA GGG AGG AGC AAA-3′	
2B-reverse	5′-TCG ATT TGC TCC TCC-3′	
c. HIVEP2 DsiRNA (Fig. 4c)		
Sense	5′-GGUCUGGAGAAACUGAUAAAGCATC-3′	
Antisense	5′-GAUGCUUUAUCAGUUUCUCCAGACCUU -3′	
d. qRT-PCR primers (Figs. 3b–d, 5)		
SLC6A3 forward	5′-ATC TAT GCG GCC TAC AAG TT-3′	2.002
SLC6A3 reverse	5′-CTC TAC ACC TTG AGC CAG T-3′	
hGAPDH forward	5′-AGG CCG GAT GTG TTC G-3′	2.017
hGAPDH reverse	5′-TTA CCA GAG TTA AAA GCA GCC-3′	
rHIVEP2 forward	5′-CTT CCT GTC CAC CTC ACT T-3′	2.027
rHIVEP2 reverse	5′-CCA GAC CTG ACC TCG TCT A-3′	
rDAT forward	5′-TCA CTC TTG GCA TTG TCC TG-3’	1.972
rDAT reverse	5′-GAG CAC GCC AAA GAG GAT AG-3′	
rGAPDH forward	5′-ATG ACT CTA CCC ACG GCA AG-3′	2.034
rGAPDH reverse	5′-TAC TCA GCA CCA GCA TCA CC-3′	
e. ChIP-PCR (Fig. 2)		
HIVEP2-DNPi forward	5′-ACA TTA TTG AAT GCT CTT AGA AG-3′	
HIVEP2-DNPi reverse	5′-GCC TCA AGA CAG ACA CTC T-3′	
hDAT + 0.79 kb forward	5′-TCG TCG GGT GTT TTA CCC AC-3′	
hDAT + 0.79 kb reverse	5′-GTG GAG GCT CTA ACA GGC AA-3′	
hACTB forward	5′-AAA GGC AAA CAC TGG TCG GA-3′	
hACTB reverse	5′-GGG ACT CAA GGC GCT AAC TG-3′	

### Cloning of TFs via Yeast One Hybrid System (Y1H)^21^

The 121-bp fragment of haplotype A^22^ was used as the search target and a human adult brain cDNA library was constructed into pP6, a pGADGH derivative plasmid, both used in Y1H in the search for proteins targeting the 121-bp (Hybrigenics Services, Paris, France). The bait construct was first checked by re-sequencing and then transformed into the yeast strain Y1H300 (MAT a, Gal4Δ, ade2-101, trp1-901, leu2-3,112, his3Δ200), integrating the DNA bait into the yeast genome. Screening was performed against the random-primed cDNA library. Clones were screened and complete cDNAs were cloned as described before^12^.

### Plasmid construction

Human HIVEP2 cDNA ORF (7.3 kb) was cloned by PfuUltra High-fidelity DNA Polymerase (Agilent Technologies) chain reaction (PCR) and then ligating the PCR product into the indicated sites in the mammalian expression vector pcDNA3.1+, resulting in the plasmid pcDNA3.1 + HIVEP2. The cloning PCR used primers for *Nhe*I (NEB)/*Not*I (NEB) sites-directed insertion. The PCR templates were cDNAs synthesized from total RNAs isolated from a combination of human SK-N-AS, IMR-32, and SH-SH5Y cells^23^. PCR fidelity was verified by DNA re-sequencing.

For luciferase (Luc) assay^13^, three reporter plasmids were generated: 1, 2A and 2B. For constructing each plasmid, two complementary single-stranded synthetic oligonucleotides were annealed together into a double-stranded oligonucleotide with *Nhe*I at the 5′ end and *Xho*I (NEB) at the 3′ end, followed by ligation of the double-stranded oligonucleotide to the same restriction sites immediately upstream of the SV40 promoter in Promega’s pGL3 Promoter Vector.

### Cell culture and transfection

SK-N-AS and BE(2)-M17 cell lines were purchased from ATCC and maintained in Dulbecco’s modified eagle’s medium (DMEM, Gibco) containing 10% fetal bovine serum (FBS, Atlanta Biologicals) and 1% Pen/Strep (Gibco). One percent non-essential amino acids (Hyclone) were added to medium for SK-N-AS. Cells were incubated at 37 ˚C in humidified air with 5% CO_2_. Cell lines were verified by PCR genotyping and tested for or removed of mycoplasma contamination (Biotool Mycoplasma Removal Kit, Bimake).

Cells were transfected chemically with plasmid DNA in Polyethylenimine (PEI, Polysciences) or lipfectamine2000. Plasmid DNA and PEI were diluted in DMEM without serum separately. The PEI solution was then added into the DNA solution, followed by vortexing for 10 s and incubation at room temperature (RT) for 15 min to form DNA-PEI complexes. At the end, the complexes were added to each well containing fresh 2% FBS growth medium. Medium was changed into fresh 10% FBS complete growth medium the next day. For 2-hexyl-4-pentynoic acid (HPA, ApexBio) treatment, HPA was added into cell medium 2 h after transfection at 5 µM/well. Sixteen hours after transfection, cells were collected for quantitative reverse transcription PCR (qRT-PCR). Three separate experiments were performed for statistical analysis at least.

### Animals

All animals, rats (alcohol preferring or P rat and alcohol non-preferring or NP rat, generation 76 and 79 from Indiana University) or male mice (C57BL/6NTac, from Taconic Biosciences, Rensselaer, New York, USA), were all adults (2–4 month old) housed under constant temperature- (21 ˚C) and humidity- (50%) on a 12 h/12 h light-dark cycle (light 07:00–19:00) with food and water available ad libitum. Rodents were housed in plastic ventilated cages. Mice housed up to five per cage, and rats housed up to two per cage. No animal was housed singly. Four individuals were used in each experiment for statistical analysis at least. All experimental procedures were approved by the Institutional Animal Care and Use Committees of McLean Hospital or the Indiana University Schools of Dentistry and Medicine (Indianapolis, IN) and were in accordance with the guidelines of the Institutional Animal Care and Use Committee of the National Institute on Drug Abuse, National Institutes of Health, and the Guide for the Care and Use of Laboratory Animals. Indicated sample size was determined by gene activity stability without randomization/blinding involved for molecular analyses.

### Immunofluorescent staining (IFS) of cells and rodent brain sections

Rabbit anti-HIVEP2 sera were kindly provided by Shunsuke Ishii of Institute of Physical and Chemical Research (RIKEN) in Japan. Methods were as described before^12^. For SK-N-AS cell staining, coverslips were blocked in 7% non-fat milk at RT for 1 h. Coverslips were incubated with rabbit ant-HIVEP2 sera (1:50 diluted in 0.01% BSA in Tris buffered saline (TBS)) or with diluted normal rabbit serum (Gibson Bioscience) as control at 4 ˚C for 48 h. The secondary antibody is Alexa Fluor 568 goat anti-rabbit IgG (1:500 diluted, RRID AB_143157, Invitrogen). Images were obtained by confocal laser scanning microscopy (Leica TCS SP8).

For mouse or rat brain section staining, adult animals were anaesthetized with 250 mg/kg Avertin (Acros Organics) by intraperitoneal (i.p.) injection. Brains were promptly collected and frozen. Before slicing, the brain was fixed in 4% PFA at 4 ˚C for 24 h and was cryoprotected in 15% (wt/v) sucrose overnight and then in 30% (wt/v) sucrose until the brain sank. After being embedded in optimal cutting temperature (OCT) compound, the frozen brain was coronally sectioned at 20 µm on a cryostat (Thermo, Microm HM 505 E). Coronal sections containing the substantia nigra and the ventral tegmental area (VTA) were selected and washed three times, 5 min each, with 0.01 M PBS for free floating staining. The sections were incubated with 0.3% Triton X-100 (diluted in PBS) for 30 min first, from the blocking step, the treatments were the same as those for cells, using mouse monoclonal anti-Tyrosine Hydroxylase (TH, 1:800 diluted in TBS containing 0.01% BSA, Millipore) for a marker of dopaminergic neurons.

### Western blotting

Western blot analyses were performed on protein fractions from cultured cells, or VTA of adult mice and rats as method described before^13^. In HIVEP2 siRNA experiment, cells were transfected with lipfectamine2000 according to the manufacturer’s instructions. 50pmol DsiRNA was applied in each well. Cells were lysed 48 h after transfection by adding 100 µL/well of RIPA buffer (Gendepot) supplemented with protease inhibitor cocktail (Millipore). Five cycles of sonication (5 s on and 5 s off) were applied for nuclear protein. Protein concentration was determined by BCA (Bicinchoninic acid) kit (Thermo Scientific) according to the manufacturer’s instruction.

For rodents, adult animals were killed by decapitation, brains were promptly collected and dissected in metal matrix (RWD). Two millimeters thick coronal brain section containing VTA between matrix 19 and 21 mm (bregma −4.52 to −6.8 mm) was cut and collected for rat brains; 1 mm thick coronal brain section between matrix 8 and 9 mm (bregma −2.92 to −3.88 mm) was cut-collected for mouse brains. VTA was sliced out of the section at one third from ventral side in the horizontal plane by removing overlying cortex and hippocampus at the periphery.

Cytoplasm and nuclear proteins of the VTA were separated using Lysis Buffer J from Cytoplasmic & Nuclear RNA purification Kit (Norgen Biotek) according to the manufacturer’s instructions. Nuclei were pelleted (4 ˚C, 12,000 × *g* for 10 min) and washed three times with cold PBS while the supernatants were collected as cytoplasm protein samples. RIPA buffer supplemented with protease inhibitor cocktail was used to lyse the nuclei as described above, the supernatants were collected as nuclear protein fractions.

Fifty µg total protein per loading well was electrophoretically separated on a 4–15% Criterion TGX precast gel (Bio-Rad). Polyvinylidene difluoride (PVDF) membranes (Santa Cruz) were incubated with primary antibody at 4 ˚C for two days. Primary antibodies used for western blots were as follows: rabbit anti-HIVEP2 sera (dilution at 1:1000), mouse anti-glyceraldehyde-3-phosphate dehydrogenase (GAPDH, 1:10,000, RRID AB_10613283, Biolegend, for cells), mouse anti-GAPDH (1:200, RRID AB_2107299, Santa Cruz, for rat brain), goat anti-RNA Pol II (1:100, RRID AB_2167471, Santa Cruz, for nuclear protein control), mouse anti-TH (1:1000, RRID AB_2201528, Millipore, for cytoplasm protein control). Amersham ECL Western Blotting Analysis System (GE Healthcare) was used to detect the primary antibody activity according to the manufacturer’s instruction. The images were captured by the Bio-Rad Molecular Imager (Bio-Rad, ChemiDOCIM XRS+) and quantitative assessment of protein bands, using the area under curve method, was executed by the Image J Software (NIH).

### Quantitative reverse transcription PCR (qRT-PCR)

For cells and rat tissues, RNA were extracted by TRIzol (Ambion), following the manufacturer’s protocol. RNA concentration was estimated with NanoDrop Lit (Thermo Fisher Scientific). Two hundred nanograms of total RNAs was reverse transcribed into cDNA by using the Verso cDNA synthesis kit (Thermo scientific) with oligo dT primers following the manufacturer’s protocol. cDNA was diluted by 10 fold with DNase-free water. cDNA samples were amplified in triplicates by incubation in the Bio-Rad CFX Connect real-time system. The amplification condition was 95 ˚C for 5 min, then for 49 cycles of 95 ˚C for 15 s, 55 ˚C for 20 s and 72 ˚C for 30 s using SsoAdvanced Universal SYBR green supermix (Bio-Rad) in a final volume of 12.5 μL, containing 1 μL of cDNA and a final concentration of 0.5 μM of forward and reverse primers. The gene of interest was then normalized against the reference gene GAPDH. The relative expression of the target gene was calculated according to the method as previously described^12^.

### Luc activity assay

Forty-eight hours after HIVEP2 overexpression or DsiRNA co-transfected with reporter constructs, cell lysates were collected for Luc activity assay and protein concentration measurements. Luc activities were measured by Promega’s Luciferase Assay System with Bio-Tek/Gen5 (Winooski). Protein assays were performed with Coomassie blue according to the manufacturer’s instructions (Thermoscientific). Briefly, 250 µL of Coomassie protein assay reagent was added to 5 µL of lysates and BSA standards. Luc activity value to protein concentration ratios were calculated for statistical analysis. Each assay was performed in duplicate.

### Chromatin immunoprecipitation (ChIP) PCR and qPCR

ChIP method was described as before^12^. Around 5 × 10^6^ BE(2)-M17 cells or 100 mg of human brain tissue were collected for ChIP assay. Tissue in 200 µL 1% formaldehyde was homogenized with a pestle mixer (Argos) and crosslinked at RT for 15 min. Independently, cells were crosslinked with 1% formaldehyde at RT for 10 min. The lysate was sonicated 12 cycles for BE(2)-M17 cells or 24 cycles for brain tissues (30 s on and 30 s off). Supernatants were transferred into new tubes and diluted 1:4 with Dilution Buffer (0.01% SDS, 1.1% Triton X-100, 1.2 mM EDTA, 16.7 mM Tris-HCl, pH 8.0, 167 mM NaCl plus the protease inhibitors) and pre-cleared with 25 µL Dynabeads Protein A (Invitrogen) at 4 ˚C for 2 h with gentle rotation. The cleared supernatant was divided into two tubes and each incubated with 10 µL anti-HIVEP2 sera or control rabbit serum at 4 ˚C for 48 h and then immunoprecipitated with 25 µL Protein A beads on a rotator at 4 ˚C for 4 h. Five hundred microliters of supernatant from the tube incubated with control rabbit IgG was collected as Input. The immunoprecipitates were washed sequentially with the following buffers: low salt buffer (0.1% SDS, 1% Triton X-100, 2 mM EDTA, 20 mM Tris-HCl pH 8.0, 150 mM NaCl), high salt buffer (0.1% SDS, 1% Triton X-100, 2 mM EDTA, 20 mM Tris-HCl pH 8.0, 500 mM NaCl), LiCl wash buffer (0.25 M LiCl, 1% Sodium deoxycholate, 1 mM EDTA, 10 mM Tris-HCl, pH 8.0), and TE (10 mM Tris-HCl, 1 mM EDTA, pH8.0). DNA was then eluted with 500 µL of elution buffer (1% SDS, 0.1 M NaHCO_3_) and the crosslinking was reversed by adding 20 μl 5 M NaCl and incubated at 65 °C overnight. 10 µL 0.5 M EDTA, 40 µL 1 M Tris-HCl (pH 6.8), and 2 µL 10 mg/mL Proteinase K were added to each sample, followed by incubation at 45 °C for 2 h to digest proteins. After purification with PCR purification kit (Promega), DNA including Input was recovered by 30 µL H_2_O and used as templates for PCR with primers to amplify the HIVEP2 binding site on *SLC6A3*. Beta-action (ACTB) was included as a negative target control. The PCR condition was 95 ˚C for 3 min, then 40 cycles of 95 ˚C for 30 s, 56 ˚C for 30 s and 72 ˚C for 30 s, followed by 72 ˚C for 7 min, using PCR Master Mix (Thermo Scientific) in a final volume of 20 μL, containing 1 µL of eluted DNA as the template. For the negative control ACTB, 0.5% DMSO was added into the PCR reaction which contained 1 µL of eluted DNA as the template. PCR products were resolved by 1.2% agarose electrophoresis and ethidium bromide-stained bands visualized by the Chemi Doc XRS Molecular Imager. qPCR was used to quantitate the binding activity.

### Secondary analysis of dbGaP GWAS datasets

Three dbGaP GWAS datasets on SUD (all past their embargo periods) and quality control have been reported before^13,24^. Imputation, association tests, logistic regression-based single nucleotide polymorphism (SNP)-SNP interactions, and meta-analyses were conducted as reported previously^13,24^. Both secondary data analysis of dbGaP GWAS datasets and the use of human post mortem brain tissue were approved by McLean Hospital Institutional Review Board (IRB) and/or National Institutes of Health (NIH).

### Statistical analysis

Data were all presented as means ± standard error of mean (SEM) where sample sizes were determined by gene activity stability. Based on GraphPad Prism (v5.04), two-way analysis of variance (ANOVA with Bonferroni post hoc tests) or Student’s *t*-test results with *p* values < 0.05 were considered as statistically significant.

## Results

By using the 121-bp of haplotype A as bait, many potential clones were obtained. However, based on in-frame fusions and adequate expression in DA neurons, HIVEP2 was chosen as a potential DAergic TF for further characterization in this study.

### HIVEP2 was present in both cytoplasm and nucleus

**Fig. 1:**
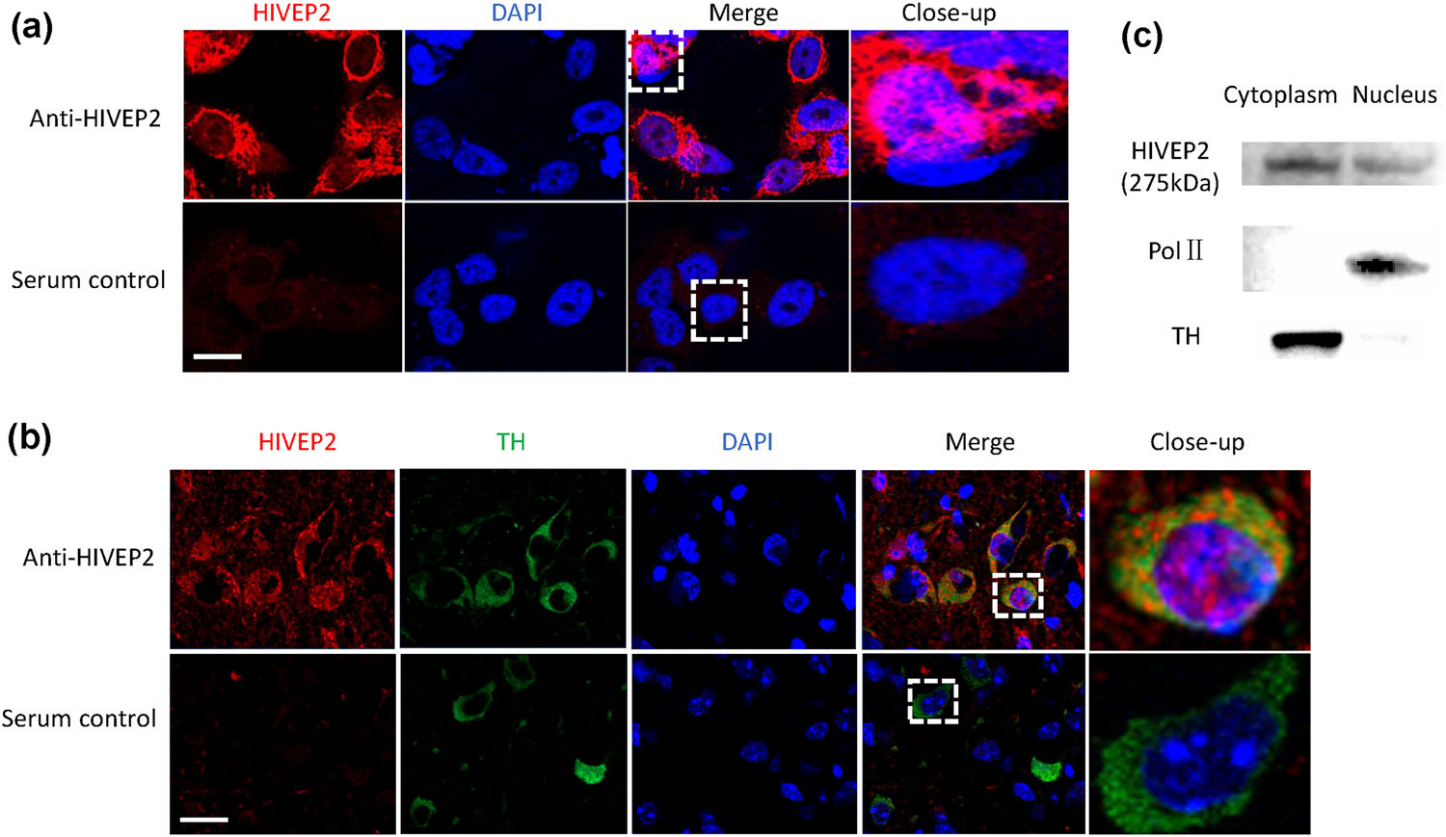
HIVEP2 protein was expressed in nuclei. **a** DAergic cell line SK-N-AS showed nuclear expression of HIVEP2. **b** DA neurons in the ventral tegmental area (VTA) of adult mouse brain showed nuclear expression of HIVEP2. Scale bar is 20 µm. **c** Western blotting showed that HIVEP2 can be detected in nuclei of mouse VTA. RNA Pol II was used as a nuclear marker and TH, a cytoplasmic marker

Immunofluorescence staining with anti-HIVEP2 sera localized this protein to both the cytoplasm and nuclei of cultured cells and DA neurons (Fig. 1a, b). In the VTA, most of the protein was expressed in DA neurons. To exclude non-specific reactivity, Western blotting detected the protein, based on its molecular weight, in both cytoplasmic and nuclear fractions of the VTA (Fig. 1c). Greater protein levels were located in the cytoplasm than in the nuclei, as determined by both in vitro and ex vivo assays.

### HIVEP2 physically bound to the 121-bp of Intron 1

**Fig. 2:**
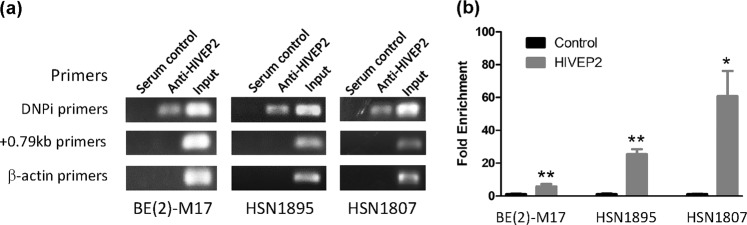
HIVEP2 bound to the promoter domain of human *SLC6A3*. **a** Regular PCR of ChIP showed that anti-HIVEP2 sera can specifically pull down the 121-bp binding domain on Intron 1 of *SLC6A3*, but neither the +0.79 kb locus which was also in Intron 1 and 1 kb away from 121-bp nor the ACTB gene. The ChIPs were done on BE(2)-M17 cell line and two human post mortem brain nigral samples: HSN1895 (a 93-year old healthy female) and HSN1807 (a 59-year old healthy male). **b** Quantification showed fold enrichment by anti-HIVEP2 sera. *p < 0.05; **p < 0.01 (ANOVA Bonferroni post hoc tests, *n* = 3)

Although this protein was cloned as a TF for the 121-bp in yeast cells, the binding activity needed to be verified in human cells. To do this three types of samples were used: the neuroblastoma cell line BE(2)-M17 of human origin as a cellular model for DA neurons, and post mortem nigral tissue from two healthy subjects, HSN1895, HSN1807. The present results from a ChIP analysis showed that anti-HIVEP2 was able to pull down the 121-bp selectively in all three samples (Fig. 2). This result confirmed the Y1H result and supported HIVEP2 as a TF in DA neurons.

### Overexpression of HIVEP2 upregulated endogenous SLC6A3 activity

**Fig. 3:**
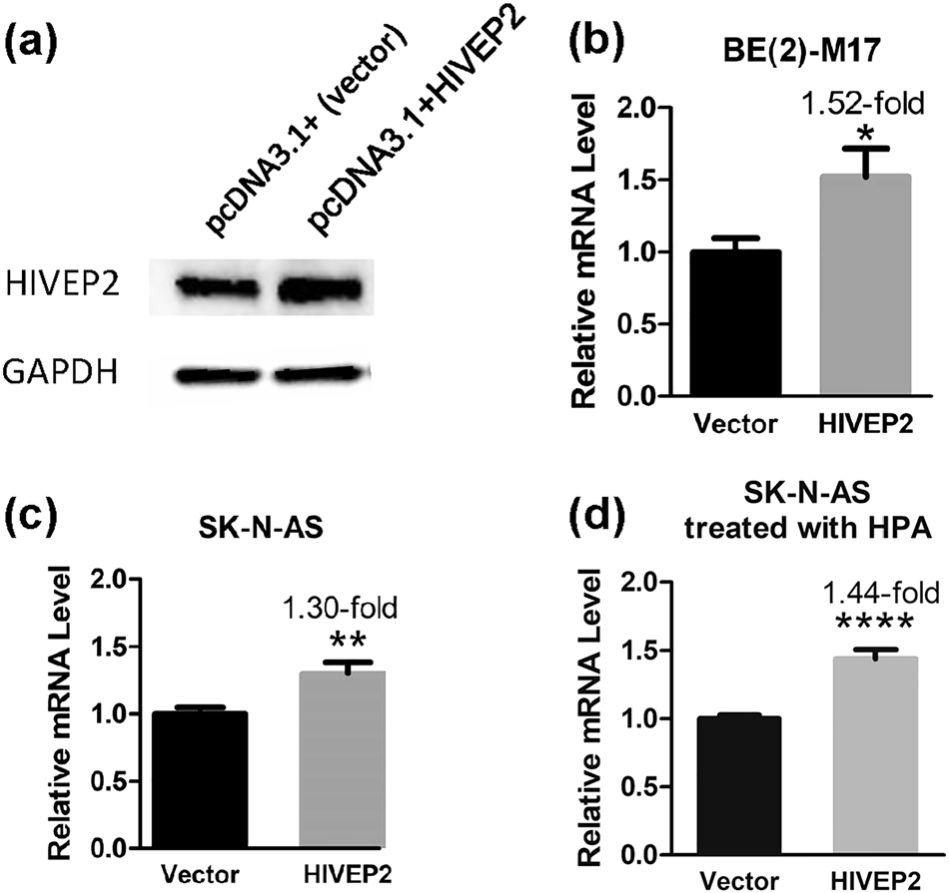
Overexpression of HIVEP2 upregulated endogenous mRNA level of *SLC6A3* in cultured BE(2)-M17 and SK-N-AS cells. **a** HIVEP2 overexpression confirmed by Western Blotting via SK-N-AS. **b** HIVEP2 overexpression increased the mRNA level of *SLC6A3* in BE(2)-M17 (*n* = 3, *p* = 0.019 by *t*-tests). **c**, **d** HIVEP2 overexpression increased the mRNA level of *SLC6A3* in SK-N-AS cells without (**c**: *n* = 3, *p* = 0.004) or with HPA 5 µm overnight treatment (**d**: *n* = 4, *p* < 0.0001)

In the human neuroblastoma SK-N-AS cells which served as another model for DA neurons^22^, overexpression of pcDNA3.1 + HIVEP2 increased the HIVEP2 protein levels as determined by Western blotting (Fig. 3a), verifying both the cDNA clone and the anti-HIVEP2 sera. Next, it was determined whether the overexpression could regulate endogenous *SLC6A3* gene activity in these cellular models. In BE(2)-M17 cells, the overexpression upregulated *SLC6A3* mRNA levels by 52% (Fig. 3b). In SK-N-AS cells, the overexpression upregulated *SLC6A3* mRNA levels by 30% (Fig. 3c); and the upregulation went up to 44% after the cells were treated with hexyl-4-pentynoic acid (HPA), an HDAC inhibitor^25^ (Fig. 3d). In this study, manipulating HIVEP2 expressions had no effect on GAPDH levels, which allowed the use of GAPDH as an internal control. These findings indicated that HIVEP2 acted as a trans-activator of *SLC6A3* in these cells, which was consistent with the cloning, the nuclear expression and the 121-bp binding results.

### HIVEP2 targeted specific DNA sequences of Intron 1

A previous study showed that GGGCCTTTCC was the binding motif or consensus sequence (CS) of HIVEP2^26^. Two homologous sequences were found in the 121-bp, “1” and “2”, which were 54 bp apart from each other. “2” contained two dinucleotide polymorphism (DNPi or rs67175440) alleles^13,27^, A and B, sharing 50–70% identity with CS (Fig. S1a: “A” defined the haplotype of the bait 121-bp). Luc-reporting analysis showed that overexpression of HIVEP2 could upregulate the SV40 promoter in a “1”- or “2”-dependent manner (Fig. S1b). The results also identified a DsiRNA molecule that could downregulate endogenous HIVEP2 protein expression (Fig. S1c). As a result, this knockdown caused Luc activity reduction to 0.63-fold via “1” as well as to 0.70-fold via “2A” and “2B”. That is, this HIVEP2 DsiRNA could downregulate the SV40 promoter in a “1”- or “2”-dependent manner. These in vitro results suggested that HIVEP2 regulates *SLC6A3* activity via binding to GTGGCTTTCT, or to a lesser extent, TTGCCTCTCC or TTGCTCCTCC, documenting the actual sequence(s) in the Intron-1 region bound by HIVEP2.

### Sex-dependent alteration of HIVEP2 expression in the P versus NP rat model

**Fig. 4:**
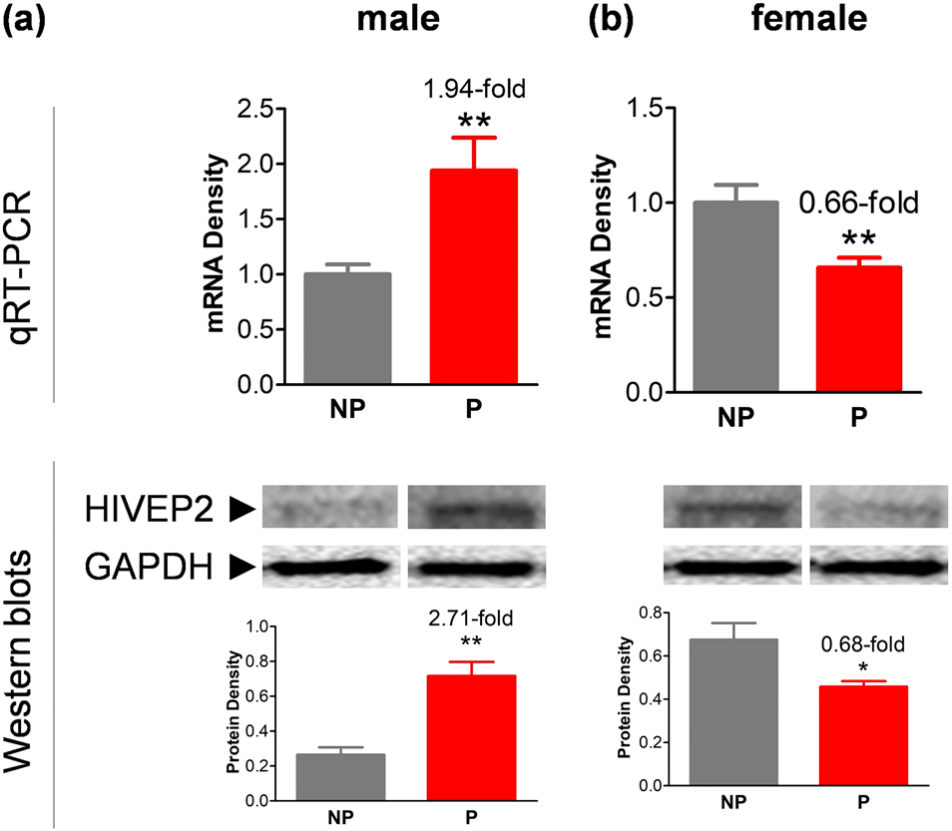
HIVEP2 expression in VTA (upper panels for mRNA, lower panels for protein) showed significant differences between NP and P rats, males (**a**) and females (**b**). Representative Western blots are shown in the middle panels and quantification, in lower panels; GAPDH was used as input controls; *t*-tests: *p < 0.05; **p < 0.01 (*t*-tests, *n* = 4–5)

P rats display excessive substance (alcohol and other drugs) taking and meet criteria proposed for a valid animal model of SUD. These rats were selected on the basis of their daily free choice intake of 10% ethanol solution vs. water, and on their preference for the alcohol solution over water. As a consequence, they also show increased responses to some other drugs of abuse^28,29^. To determine potential association between *HIVEP2* and SUD, HIVEP2 activity was investigated in the VTA of naive P *vs* NP rats, an animal model of DAergic pathology^30–34^, using both males and females. The results showed sex-dependence, with activity at both mRNA and protein levels being significantly higher in male P rats compared with male NP rats, whereas significantly lower in female P rats compared with female NP rats (Fig. 4). We also have looked at two other recently cloned TFs, SRP54, and Nfe2l1^12^, and found that neither showed >50% changes in the males or any significant changes in the females (protein data not shown here), indicating a selective role of HIVEP2 in the genomic etiology of SUD. These data supported our hypothesis that HIVEP2 can confer a risk to develop SUD, although an important finding was that this effect appeared to be sex-dependent.

### Significant interactions and large effect size epistasis between HIVEP2 and SLC6A3 in SUD

**Fig. 5:**
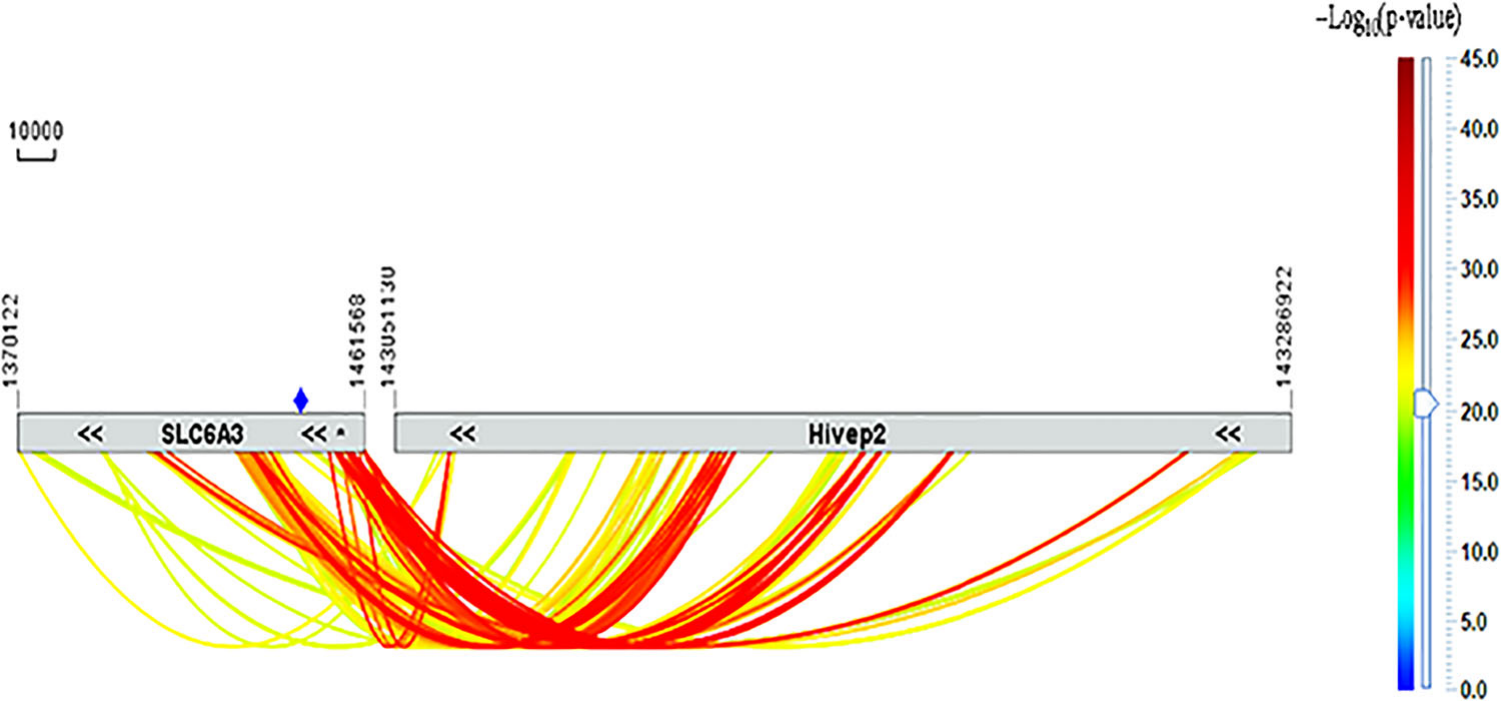
Human genetics of SUD via SNP interactions (colored curves) between *HIVEP2* (on the right) and *SLC6A3* (on the left; 121-bp: location indicated by blue diamond). Data are from meta-analysis of logistic regression results by using three dbGaP datasets of SUD (alcohol and cigarette smoking). Gray horizontal bars, on the left is *SLC6A3* in chr5 and on the right is *HIVEP2* in chr6 with coordinates indicated above the bars; double black arrow symbols, location of transcription start and ending sites (both genes run on the minus strands of the chromosomes); thermometer bar on the right, interaction strength *(p-*value from meta-analysis) in the form of –Log_10_*(p-*value); only shown are those reaching absolute genome-wide significance (*p* < 10^−20^); chromosomal scale: 10 kb

To validate a clinical relevance of the significant findings in the rat model, we consulted with the dbGaP GWAS datasets from four cohorts with SUD, as described before^13^. Based on meta-analysis of logistic regression case-control association results from the four cohorts of unrelated individuals, we have found that there are >1200 significant including more than 300 absolute genome-wide significant interactions between *HIVEP2* and *SLC6A3* in the genetic etiology of SUD (Fig. 5, top ones detailed in Table S1; “absolute” means Bonferroni correction by all base-pairs of the human genome). These interactions are much more significant than the reported ones between the downregulator’s gene ^AZI2^3’UTR and *SLC6A3*^13^. For example, rs12525545 in Intron 3 of *HIVEP2* interacted with rs748209 located in a distal (−12 kb) promoter region of *SLC6A3* with a *p*_meta_ value of 1.12 × 10^−37^ and an odds ratio_meta_ of 0.16. However, there were no significant main effects, that is, allelic associations between *HIVEP2* and SUD (Table S2), with males showing stronger signals than females (details not shown here). There appears to be three rather distinct regions in *SLC6A3* where the interactions occur but none of previously used markers were located within the two CSs so that the epistatic signals came likely from linkage disequilibrium.

## Discussion

The present study identified HIVEP2, as a new DAergic TF, and has demonstrated that it binds to *SLC6A3* and regulates *SLC6A3* activity, which is the hallmark of DAergic neurons. The findings also revealed that altered *HIVEP2* activity may be associated with vulnerability for developing SUD; such that altered expression levels were associated with this phenotype in the P vs NP animal model of SUD (alcohol, nicotine, and cocaine)^30–34^. There seem substantial sex differences in ethanol responses in P rats. It has been reported that the males show significantly higher preference to 8% ethanol than the females during a period of 90 days^35^. We have also observed that in an adolescent binge drink model, the males show higher consumption than the females^36^. These behavioral data fit with our finding that elevated HIVEP2 expression in males be a risk factor for AUD in males. Due to limitations of cultured assay systems and of manipulating the large HIVEP2 gene (10 kb), these in vitro results did not necessarily represent the in vivo effect sizes but the functional human molecular genetic findings may help delineate disease mechanisms in humans.

Previous study showed that the 121-bp of haplotype A, especially a 36 bp subfragment containing the HIVEP2 target “1”, displayed inhibitory activity only on the SV40 promoter^22^. It was an expectation that we would be able to clone a *trans*-repressor, instead of a *trans*-activator (HIVEP2). Therefore, it remains unknown what mediated the inhibitory activity of 121-bp. One possibility was that the long non-coding RNA (lncRNA) ^AZI2^3’UTR was contributing to the inhibitory activity. Another possibility was that the activation-based Y1H system was unable to clone such as a repressor so that other approaches might need to be considered.

HIVEP2 (also known as Schnurri-2) was initially cloned as a zinc finger protein binding to the human immunodeficiency virus type (HIV-1) enhancer and to an enhancer of major histocompatibility complex class I genes^26,37^. This category of TFs are supposed to be expressed primarily in the nuclei but our data suggest that they also are localized to the cytoplasm with unknown function. Lately, this protein has been related to brain function. The gene knockout mice display hyperactivity^38^, consistent with a view that HIVEP2 is an activator of *SLC6A3*. These mice also display schizophrenia-related phenotypes, increased anxiety, intellectual disability, and memory deficits^39–43^. However, until the present study its activity has not been tested in the context of a vulnerability to develop SUD.

The epistatic association significances were much larger than those for ^AZI2^3'UTR^13^ but it remains unclear where the epistasis signals came from in the meta-analysis of case-control associations with SUD (also alcohol, nicotine and cocaine). The HIVEP2 target sequences in the 121-bp carried two known polymorphisms, DNPi (rs2937640 and rs2975223) and rs28382220, both were located in the “2” sequences which may have contributed to some of the observed significant epistasis but none of these SNPs were included in the previous GWAS datasets; target sequence “1” carried no known polymorphism. Nevertheless, there was the possibility of “synthetic associations” in the observed associations^44,45^. Based on the epistasis, HIVEP2 could bind to other loci including the distal promoter regions of *SLC6A3*.

A main limitation of this study is that the observed associations do not necessarily indicate causation. To address this, protein-DNA binding activity and associated phenotypes related to SUD could be studied in gene knockout mice. Another unknown is the level of *SLC6A3*-wide, or even genome-wide, binding activity of HIVEP2 in DA neurons. In addition, gene and/or protein activity has not been studied comprehensively in a brain-region-specific manner. Nor has its function been studied in the cytoplasmic fraction. These limitations warrant further investigations.

## Conclusions

HIVEP2 is a new DAergic TF expressed in both cytoplasm and nuclei. Altered HIVEP2 activity in the VTA is associated with SUD in a rat model; significant *HIVEP2-SLC6A3* epistasis is found for SUD in humans. These data suggest that regulated HIVEP2 in DA neurons of reward pathways may modulate vulnerability to develop SUD.

## Supplementary information

Supplemetary
