# Supplementary material for: Identification of HIVEP2 as a dopaminergic transcription factor related to substance use disorders in rats and humans
Source: Transl Psychiatry. 2019 Oct 4;9:247. doi: 10.1038/s41398-019-0573-8 (PMC6778090; doi:10.1038/s41398-019-0573-8)
Supplement: Supplementary file 1 — Supplemetary [file 41398_2019_573_MOESM1_ESM.pdf]

(a)

```
CS:  GGGCCTTTCC Identity
 1:  GtGgCTTTCt 7/10
2A:  ttGCCTcTCC 7/10
2B:  ttGCtccTCC 5/10
```

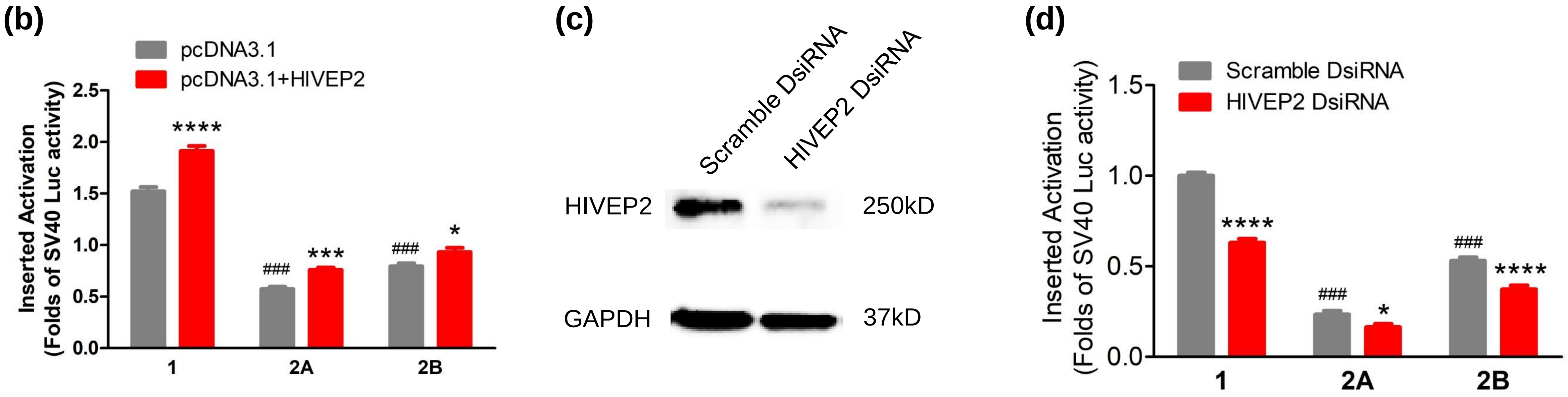

Fig. S1 HIVEP2 overexpression (**b**) or knockdown by a DsiRNA (**c**, **d**) could increase or inhibit target sequence-based SV40 promoter activity. (**a**) HIVEP2 consensus sequence (CS) and its three matched sequences in 121-bp. Identity: comparing to CS; lower case, mismatch; red, two alleles of DNPi, A or B. "1" was 54 bp upstream of "2A" or "2B" (towards Exon 1). (**b**) HIVEP2 overexpression increased the sequences-based promoter activities (*n*=4 independent cell culture preparations). (**c**) HIVEP2 DsiRNA knocked down protein expression in SK-N-AS cell line based upon Western Blotting (this knockdown was confirmed by qRT-PCR analysis of mRNA). (**d**) HIVEP2 DsiRNA decreased the sequences-based promoter activity (*n*=4 independent cell culture preparations). * means differences between treatment and control; #, difference between target sequences in the control condition: one symbol for $p<0.05$, three for $p<0.001$ and four for $p<0.0001$ by ANOVA tests. The small effect sizes were likely due to the ineffectiveness of working with or on the large HIVEP2 gene (10 kb) in double transfections.

Table S1: Epistasis between *HIVEP2* (chr6) and *SLC6A3* (chr5) in SUD by meta-analysis.

(Only top 100 out of 1258 significant ones[#] are shown here)

HIVEP2 in chr6		*SLC6A3* in chr5			
**ID2**	**BP2**	**ID1**	**BP1**	*p* (R)	OR(R)
rs62432361	143197351	rs4738	1461568	7.21E-39	0.15
rs62430711	143178455	rs4738	1461568	8.69E-39	0.15
rs12525545	143174387	rs4738	1461568	8.69E-39	0.15
rs12525545	143174387	rs748209	1457554	1.12E-37	0.16
rs62430711	143178455	rs748209	1457554	1.12E-37	0.16
rs62432361	143197351	rs905201	1457986	1.12E-37	0.16
rs62432361	143197351	rs748209	1457554	1.14E-37	0.16
rs62430711	143178455	rs905201	1457986	1.33E-37	0.16
rs12525545	143174387	rs905201	1457986	1.33E-37	0.16
rs62432361	143197351	rs12652860	1453772	1.88E-37	0.16
rs62430711	143178455	rs2937650	1458018	2.22E-37	0.16
rs12525545	143174387	rs2937650	1458018	2.22E-37	0.16
rs62430711	143178455	rs12652860	1453772	2.23E-37	0.16
rs12525545	143174387	rs12652860	1453772	2.23E-37	0.16
rs62432361	143197351	rs2937650	1458018	2.29E-37	0.16
rs62430711	143178455	rs1478435	1454612	3.58E-37	0.16
rs12525545	143174387	rs1478435	1454612	3.58E-37	0.16
rs62432361	143197351	rs1478435	1454612	3.70E-37	0.16
rs62432361	143197351	rs10060889	1456803	4.38E-37	0.16
rs62430711	143178455	rs10060889	1456803	5.07E-37	0.16
rs12525545	143174387	rs10060889	1456803	5.07E-37	0.16
rs111574947	143140093	rs456774	1432202	6.64E-33	11.31
rs75009943	143138145	rs456774	1432202	2.11E-31	9.20
rs62430711	143178455	rs456774	1432202	1.68E-30	6.58
rs12525545	143174387	rs456774	1432202	1.68E-30	6.58
rs79908324	143136407	rs456774	1432202	1.71E-30	8.85
rs111822952	143134907	rs456774	1432202	1.71E-30	8.85
rs111574947	143140093	rs4738	1461568	2.55E-30	0.13
rs79908324	143136407	rs4738	1461568	2.47E-29	0.16
rs111822952	143134907	rs4738	1461568	2.47E-29	0.16
rs7765677	143259563	rs11564763	1406663	4.63E-29	22.34
rs111574947	143140093	rs905201	1457986	5.76E-29	0.14
rs12333188	143065590	rs11750173	1460131	6.02E-29	0.01
rs12333188	143065590	rs11747778	1452193	6.79E-29	0.01
rs111574947	143140093	rs12652860	1453772	8.29E-29	0.14
rs111574947	143140093	rs10060889	1456803	1.50E-28	0.14
rs75009943	143138145	rs4738	1461568	2.00E-28	0.16
rs111574947	143140093	rs428280	1436476	2.77E-28	9.15
rs7765677	143259563	rs2245660	1409719	5.53E-28	21.29
rs79908324	143136407	rs10060889	1456803	1.13E-27	0.17
rs111822952	143134907	rs10060889	1456803	1.13E-27	0.17
rs79908324	143136407	rs905201	1457986	1.29E-27	0.16
rs111822952	143134907	rs905201	1457986	1.29E-27	0.16
rs62432361	143197351	rs456774	1432202	1.59E-27	6.87

rs12333188	143065590	rs34813657	1455482	1.99E-27	0.01
rs111574947	143140093	rs465989	1432881	2.92E-27	8.68
rs111574947	143140093	rs458609	1434306	2.95E-27	8.68
rs75009943	143138145	rs905201	1457986	3.23E-27	0.17
rs111574947	143140093	rs461753	1431214	3.47E-27	8.79
rs113328833	143127371	rs456774	1432202	3.99E-27	7.06
rs7765677	143259563	rs11564764	1405806	5.16E-27	19.13
rs111574947	143140093	rs393795	1428514	5.67E-27	8.58
rs111574947	143140093	rs638964	1433867	6.17E-27	8.53
rs111574947	143140093	rs460700	1429969	6.43E-27	8.57
rs111574947	143140093	rs456082	1430515	7.23E-27	8.50
rs111574947	143140093	rs638577	1433831	7.23E-27	8.50
rs111574947	143140093	rs487781	1433931	7.23E-27	8.50
rs111574947	143140093	rs250682	1427803	7.31E-27	8.50
rs111574947	143140093	rs461677	1431992	7.82E-27	8.49
rs111574947	143140093	rs459141	1432042	7.82E-27	8.49
rs111574947	143140093	rs460000	1432825	7.82E-27	8.49
rs111574947	143140093	rs464061	1430244	7.91E-27	8.49
rs111574947	143140093	rs410209	1430775	7.91E-27	8.49
rs111574947	143140093	rs458860	1430933	7.91E-27	8.49
rs111574947	143140093	rs57106193	1431123	7.91E-27	8.49
rs111574947	143140093	rs463379	1431164	7.91E-27	8.49
rs111574947	143140093	rs458632	1431302	7.91E-27	8.49
rs111574947	143140093	rs460934	1431306	7.91E-27	8.49
rs111574947	143140093	rs457702	1434430	7.99E-27	8.49
rs111574947	143140093	rs465130	1432876	8.67E-27	8.49
rs75009943	143138145	rs10060889	1456803	8.79E-27	0.17
rs111574947	143140093	rs460007	1431853	1.37E-26	8.42
rs62432361	143197351	rs461753	1431214	2.28E-26	5.74
rs111574947	143140093	rs462053	1431554	3.95E-26	8.25
rs75156945	143128320	rs456774	1432202	4.26E-26	6.98
rs111574947	143140093	rs409588	1430834	8.89E-26	8.58
rs62432361	143197351	rs460007	1431853	1.02E-25	5.53
rs75009943	143138145	rs428280	1436476	1.79E-25	7.10
rs111574947	143140093	rs427284	1429187	1.88E-25	8.05
rs62432361	143197351	rs638964	1433867	2.18E-25	5.46
rs62432361	143197351	rs456082	1430515	2.61E-25	5.45
rs62432361	143197351	rs638577	1433831	2.61E-25	5.45
rs62432361	143197351	rs487781	1433931	2.61E-25	5.45
rs80170979	143122021	rs456774	1432202	2.64E-25	6.64
rs75603838	143121863	rs456774	1432202	2.64E-25	6.64
rs62432361	143197351	rs250682	1427803	2.65E-25	5.44
rs62430711	143178455	rs460007	1431853	2.86E-25	5.44
rs62432361	143197351	rs461677	1431992	2.86E-25	5.44
rs62432361	143197351	rs460000	1432825	2.86E-25	5.44
rs62432361	143197351	rs464061	1430244	2.90E-25	5.44
rs62432361	143197351	rs410209	1430775	2.90E-25	5.44
rs62432361	143197351	rs458860	1430933	2.90E-25	5.44

rs62432361	143197351	rs57106193	1431123	2.90E-25	5.44
rs62432361	143197351	rs463379	1431164	2.90E-25	5.44
rs62432361	143197351	rs458632	1431302	2.90E-25	5.44
rs62432361	143197351	rs460934	1431306	2.90E-25	5.44
rs62432361	143197351	rs457702	1434430	2.95E-25	5.44
rs7765677	143259563	rs11564766	1404726	3.77E-25	15.67
rs9386015	143272689	rs11564763	1406663	4.87E-25	12.97
rs17071986	143066785	rs34813657	1455482	5.54E-25	0.01

# Bonferroni cut-off *p* value: 4.33359E-11

Table S2. Allelic association between *HIVEP2* and SUD by meta-analysis.

BP	SNP	*p* (R)	OR(R)
143263289	rs6933661	0.000340	1.20
143270216	rs2095369	0.000646	1.18
143278739	rs6922444	0.000839	1.13
143269509	rs1467817	0.000876	1.17
143268110	rs9399414	0.001244	1.17
143286922	rs11155270	0.001270	0.87
143285530	rs9484681	0.001327	1.15
143075242	rs197477	0.001653	0.87
143277615	rs6936523	0.001940	1.15
143276295	rs11751124	0.001961	0.87
143058692	rs6570526	0.001962	0.88
143057067	rs4895598	0.001966	0.88
143279175	rs13203900	0.002164	0.88
143219105	rs6900703	0.002244	1.12
143279014	rs6900153	0.002337	1.15
143197305	rs198679	0.002422	0.89
143197591	rs198680	0.002579	0.89
143227412	rs4896604	0.002593	1.12
143228163	rs6570533	0.002593	1.12
143180726	rs62430713	0.002605	0.86
143194288	rs198676	0.002745	0.89
143225845	rs9390032	0.002837	0.81
143220180	rs9496475	0.003090	1.11
143061297	rs197491	0.003134	0.87
143192180	rs198671	0.003173	0.89
143192734	rs198673	0.003206	0.89
143074700	rs169098	0.003238	0.87
143193971	rs72995085	0.003291	0.87
143055614	rs3900201	0.003302	0.87
143274232	rs6925124	0.003313	1.14
143073041	rs3383	0.003371	0.88
143056556	rs9390022	0.003472	0.88
143075614	rs197476	0.003477	0.88
143060909	rs197493	0.003490	1.15
143228935	rs4896605	0.003495	1.11
143188746	rs198669	0.003532	0.89
143188910	rs198670	0.003532	0.89
143058002	rs57584479	0.003579	0.88
143058122	rs4896592	0.003632	0.88
143228053	rs6570532	0.003645	1.11
143187926	rs107118	0.003829	0.89
143075829	rs197475	0.004165	0.88
143050996	rs56146803	0.004178	0.88
143065213	rs117698511	0.004365	1.78
143059945	rs4896593	0.004529	0.88

143082403	rs137976777	0.004612	0.50
143192850	rs198674	0.005214	0.90
143187857	rs107119	0.005442	0.90
143055561	rs683831	0.005472	1.14
143219367	rs35146504	0.005502	0.82
143219671	rs13207993	0.005502	0.82
143275398	rs9496486	0.005521	1.13
143061160	rs197492	0.005537	1.15
143222770	rs13190880	0.005652	0.82
143224084	rs13195529	0.005676	0.82
143083063	rs638880	0.006186	1.11
143276995	rs9484680	0.006410	1.14
143234506	rs9376716	0.006944	0.83
143238285	rs9390037	0.006944	0.83
143054384	rs7349872	0.007060	0.89
143235840	rs62428765	0.007276	0.83
143080385	rs499166	0.007367	1.11
143228641	rs9403420	0.007766	0.84
143052398	rs802625	0.007773	1.14
143234975	rs34431998	0.007833	0.83
143224510	rs9390030	0.007862	0.84
143224786	rs9403416	0.007862	0.84
143224993	rs9403417	0.007862	0.84
143225220	rs9403418	0.007902	0.84
143225425	rs9390031	0.007902	0.84
143225904	rs9390033	0.007902	0.84
143227202	rs9399410	0.007902	0.84
143233711	rs36091009	0.007929	0.83
143233782	rs36072087	0.007929	0.83
143054622	rs687904	0.007962	1.14
143224110	rs9390029	0.007999	0.84
143112816	rs184727064	0.008002	0.55
143230355	rs75486200	0.008063	0.83
143231477	rs34616428	0.008063	0.83
143237975	rs62428766	0.008148	0.83
143235290	rs62428764	0.008245	0.83
143231149	rs9403421	0.008691	0.84
143057185	rs655648	0.009307	1.14
143053893	rs12192768	0.009669	0.89
